# Birthweight percentiles for twin birth neonates by gestational age in China

**DOI:** 10.1038/srep31290

**Published:** 2016-08-10

**Authors:** Bin Zhang, Zhongqiang Cao, Yiming Zhang, Cong Yao, Chao Xiong, Yaqi Zhang, Youjie Wang, Aifen Zhou

**Affiliations:** 1 Wuhan Medical & Healthcare Center for Women and Children, Wuhan, China; 2 Department of Maternal and Child Health, School of Public Health, Tongji Medical College, Huazhong University of Science & Technology, Wuhan, China

## Abstract

Localized birthweight references for gestational ages serve as an essential tool in accurate evaluation of atypical birth outcomes. Such references for twin births are currently not available in China. The aim of this study was to construct up-to-data sex specific birth weight references by gestational ages for twin births in China. We conducted a population-based analysis on the data of 22,507 eligible living twin infants with births dated between 8/01/2006 and 8/31/2015 from all 95 hospitals within the Wuhan area. Gestational ages in complete weeks were determined using a combination of last-menstrual-period based (LMP) estimation and ultrasound examination. Smoothed percentile curves were created by the Lambda Mu Sigma (LMS) method. Reference of the 3^rd^, 10^th^, 25^th^, 50^th^, 75^th^, 90^th^, 97^th^ percentiles birth weight by sex and gestational age were made using 11,861 male and 10,646 female twin newborns with gestational age 26–42 weeks. Separate birthweight percentiles curves for male and female twins were constructed. In summary, our study firstly presents percentile curves of birthweight by gestational age for Chinese twin neonates. Further research is required for the validation and implementation of twin birthweight curves into clinical practice.

Several population-based studies have documented a strong temporal increase in the frequency and rate of multiple births^1^,^2^. Currently, twin births account for 2–4% of all births worldwide^3^,^4^,^5^. Twin pregnancies, in comparison with singletons, are at higher risk of fetal anomalies^6^, preterm births^7^, and perinatal morbidity and mortality, as well as long-term disability^8^,^9^,^10^, largely because of prematurity and fetal growth restriction^11^. The optimal fetal growth in twins remains inadequately defined^12^.

The average birthweight of twins is significantly lower than singletons beginning around week 30 to 32^13^. Thus, if the growth trajectory of twins is followed on a singleton reference, the normal deceleration of growth in twins may be interpreted as pathological slowing^3^,^10^,^14^. Furthermore, increasing evidences that optimal birth weights are lower for twins than for singletons support the use of different reference charts to assess the growth of twins^15^. Therefore, many efforts have been made by researchers toward developing twin-specific growth curves.

So far, birth weight standards for twins have been published and updated in several countries including Argentina, Australia, Canada, Japan, Norway and Taiwan^11^,^16^,^17^,^18^,^19^. By contrast, this reference has not yet been established in China. In this study, we attempted to construct up-to-date, gestational age specific birth weight references based on data from a population-based, local birth surveillance system in Wuhan, a city located in center China.

## Results

The mean gestational age and birthweight were calculated according to sex, sex combination within the twin pair, method of delivery, birth order, parity, presence of preterm birth, and low birthweight as represented in Table 1. Having a male, unlike-sexed pairs, twins delivered by caesarean section, or first born twin individuals were found to have heavier birthweight. Females and caesarean section delivery twins had longer mean gestational ages. Over half of the twins were preterm or exhibited low Birthweight.

**Figure 1:**
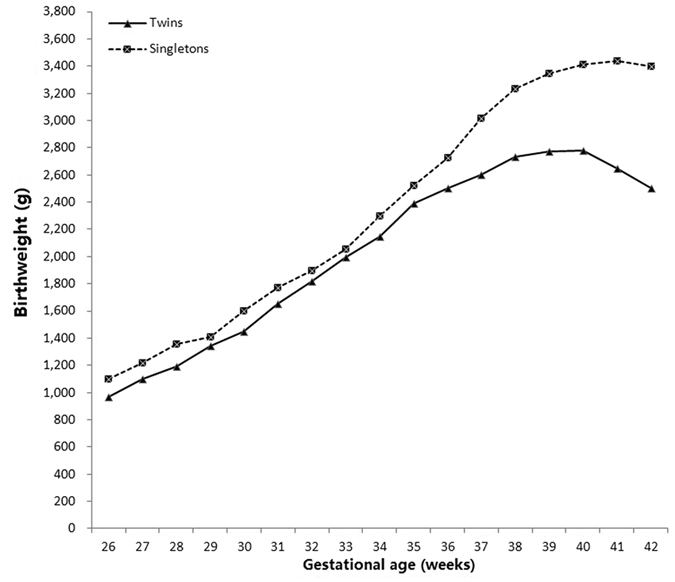
Comparison of 50^th^ percentile of birthweight by gestational age from 26–42 weeks between living singleton (n = 808, 889) and twin births (n = 22,507) registry during 2006–2015 in Wuhan, China.

Figure 1 shows a comparison of the 50^th^ percentile of birthweight by gestational age between singletons and twins. For all gestational ages twins births had significantly lower birthweight than singletons (*P* < 0.001). This difference increased with increasing gestational age from 32 weeks. The minimum average divergence of birthweight was 58 grams at 32 weeks, while the maximum divergence of birth weight was 900 grams at 42 weeks.

**Figure 2:**
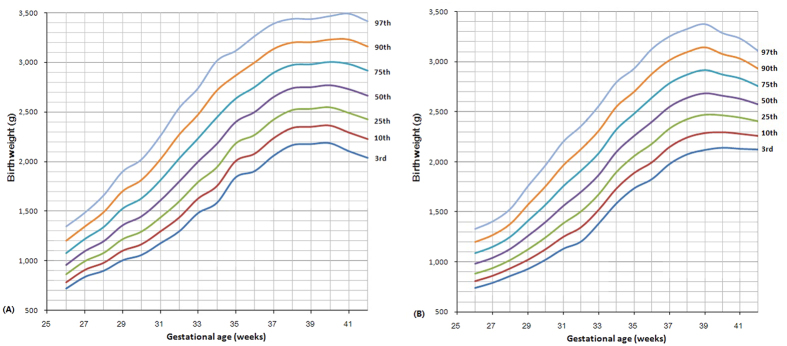
The percentile curves of birth weight by gestational age for male (**A**) and female (**B**) twin newborns in Wuhan, China.

Smoothed reference curves for birthweight by gestational age of the 3^rd^, 10^th^, 25^th^, 50^th^, 75^th^, 90^th^, and 97^th^ percentiles for male (A) and female (B) live born twins are shown in Fig. 2. The LMS parameters for the birthweight curves and the value of cutoffs for 3^rd^, 10^th^, 25^th^, 50^th^, 75^th^, 90^th^, and 97^th^ birthweight percentiles by gestational age for female and male twins were presented in Tables 2 and 3, respectively. At all gestational ages, the median birthweight of male twins were higher than that of females, and the average difference through each gestational week was about 83.7 grams.

**Figure 3:**
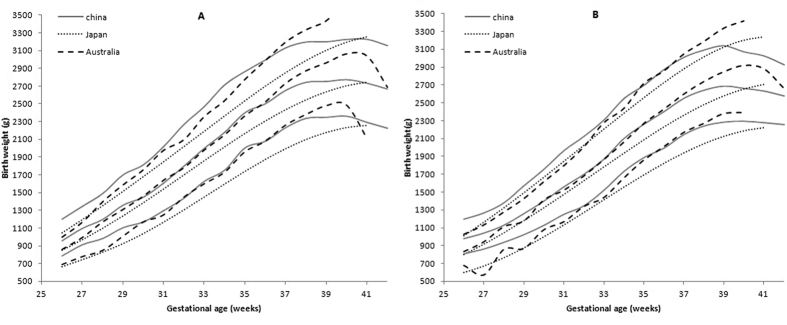
The percentile curves of Wuhan birth weight (black solid line) compared with curves of Australian (dashed line) and Japanese (dot line) for male (**A**) and female (**B**) twin newborns. The line represents 10th, 50th and 90th birth weight percentiles, respectively.

We also compared the reference curves of birthweight for Wuhan twins with that of Japan^20^ and Australian^17^ twins (Fig. 3A,B). Overall, the 10^th^ and 50^th^ percentile curves of Wuhan twins were similar to the Australian curves until at the 36^th^ week of gestational age in both sex, and the 90^th^ birthweight percentile of Wuhan twins shows slightly heavier than that of Australian twins from 26 to 36 weeks. However, the Wuhan curves are significant below Australian curves after 37 weeks of gestational age. Meanwhile, the 10^th^, 50^th^, and 90^th^ percentiles birthweight of the Japan curve were significantly lower than that of Wuhan’s from 26–40 weeks of gestational age in both sexes.

## Discussion

This paper presented sex-specific twin birthweight references between 26 and 42 weeks of gestation, based on a cross-sectional study of city-wide population in Wuhan, China, during 2006–2015. This study is the first to provide gestational-age-specific weight references for twins in the center China area. Although this study concerned primarily with the Wuhan population, these references should be considered meaningful for a majority of twin infants in the center China provinces (specifically, Hunan, Anhui, Henan, Jianxi, Chongqin), due to the homogeneity of their ethnic (predominantly Han), economic, geographical, and other features that had potential impact on birthweight distribution. Since more than 99.0% of all living twin births in Wuhan during that period were included in this analysis, we believe our data can represent the entire Wuhan population. Thus, these gestational-age-specific birthweight percentile charts are helpful as references for neonatal health care professionals and researchers to identify the high risk neonates who require more intensive observation and special care. However, it is important to note that percentile charts do not represent fetal growth standards in utero but weight at birth^21^.

Several population-based studies globally have documented a strong increase in the frequency and rate of multiple birth^2^,^22^. In particular, the rate of twinning has increased by 75% over the last 3 decades in the US^23^. Similar temporal increases in the rate of twinning have been documented in other industrialized countries, including Canada (from 18.0 per 1000 in 1981 to 23.1 per 1000 in 1997-a relative increase of 28%), England and Wales (from 19.4 per 1000 in 1982 to 27.3 per 1000 in 1997-a relative increase of 41%), and France (from 19.5 per 1000 in 1982 to 28.3 per 1000 in 1997-a relative increase of 45%)^24^. Data from other industrialized countries, including Australia, Austria, Finland, Japan, Norway, Singapore, Sweden, have also shown similar increases in the rate of twinning^25^. Lastly, the incidence of twins increased from 0.6% during 1954–1964 to 2.5% from 1998 to 2002 in Taiwan^26^. Our study also found a strong increase in the frequency and rate of twin birth (from 18.0 per 1000 in 2006 to 39.0 per 1000 in 2015-a relative increase of 1.16 times). Considering this increasing trend of twin births and the absence of a localized birthweight reference for twin neonates, we developed these specific twin birth weight curves. These curves will be particularly useful for assessment of birth weight of twin births.

When compared with their singleton counterparts, the mean birthweight for twins falls below that of singletons at every gestational age. However, this difference is small up to 35 weeks (an average divergence of <130 g). From 35 weeks of gestation, the mean birthweight for twins falls increasingly below that of singletons. Our result is comparable to other studies that also reported similar findings. A population-based study of twins from England showed that the divergence may occur as early as 24 weeks of gestation; a time at which singletons were 100 gram heavier than twins on average^27^. Another register-based study^28^ and two large population-based studies in US^13^ and Norway^11^ showed that the divergence occurred from the gestational age of 30 weeks. However, another study by Naeye *et al*.^29^ revealed that the divergence occurred from 33 weeks, while Min *et al*.^30^ did not find any significant difference between the birthweight of twins and singletons until 36 weeks of gestation. Additionally, our data indicated that birthweight for twins peaked at 40 weeks and then declined 2 weeks earlier than the peak birthweight for singletons. The greatest divergence in mean birthweight was at 42 weeks, which was 900 gram below singletons. The discrepancy of birthweight for twins and singletons after a certain weeks of gestation (i.e. 35 weeks in our study) might be a reflection of the negative in-utero conditions such as intrauterine constraint and/or placental insufficiency, which further resulted in accelerated physiological maturation^21^,^31^. If so, the earlier birthweight peak in twins than singletons would make sense.

It is well known that there are racial and ethnic differences in birthweight. A comparison of twin birthweight data from Australia, United States, The Netherlands, Japan, and South Korea showed the total phenotypic variances of birthweight were about 45% larger in Caucasians than in East Asians. The largest phenotypic variances were mainly attributable to a greater shared environmental (include gestational age, the physical and physiological characteristics of the uterine environment, and the genotype of the mother who provides the uterine environment) variance of birthweight in Caucasians (ranging from 62% to 67% of variance) than Asians (48% to 53%)^32^. These findings underline the importance of developing ethnicity specific references for birthweight.

So far, reference birthweight was also developed from population-based data in other countries such as Norway, Australia, Belgium, Israel, and Taiwan etc^11^,^17^,^26^,^33^,^34^. To understand the difference of the birthweight distribution between Asian and Caucasians twins, we compared the 10^th^, 50^th^, and 90^th^ birthweight percentile curves of twins made by our data to those of Asian twins (i.e. Japanese) and Caucasian twins (i.e. Australian). When compared with Australian reference of percentile curves, we found the Chinese reference value were slightly higher in preterm twins but significantly lower in term twins. However, when compared with Japanese reference, all the 10^th^, 50^th^ and 90^th^ percentile reference values of Chinese twin were completely higher than that of Japanese twins in both sexes. The data that used to produce the percentiles of birthweight in other studies were not update to the same time period as in our study. This difference in time period when the measurements were taken may be a main contribution to the differences observed between our study and studies from other regions. For example, the socioeconomic status in difference periods is one of the major contributors to the variation of newborns’ birthweight over time^35^. Another factor that could account for the differences between our and other studies is race-specific influences as discussed above^32^. Therefore, the references of birthweight percentiles by gestational age should be formulated while keeping racial differences in mind and should be updated every 5–10 years.

In this study, we set strict exclusion criteria and excluded extreme outliers. The abnormal birthweight of malformed fetuses or stillbirths may indicate an early death with a birthweight not corresponded to the gestational age. For some births, reported gestational age and birthweight combinations were implausible, especially very high birthweight recorded for some preterm infants. Those exclusion criteria enable a fair comparison with other current birthweight standards, most of which are based on livebirths, and provide birthweight references for the risk assessment of neonatal and infant mortality and morbidity.

There are some limitations in the present study. Firstly, the data in WBRIS were obtained from different centers which probably added an inter-center variance. Furthermore, Birth records in this study were collected within a long period. The environment of pregnant women including socioeconomic status, diet and nutrition had a remarkable improvement in Wuhan from 2006 to 2015. During the last decade, the rate of twining pregnancy had increased from 1.8% to 3.9%, while the average maternal age at childbirth was raised from 27.3 (4.7) year old to 30.1 (5.7) year old. These changes indicated that twin births due to assisted conception are becoming more popular. Besides these changes, the rate of preterm birth had also increased from 38.7% to 62.0%. On the other hand, the prevalence of neonatal death (from 1.5% to 0.4%) and stillbirth (from 3.5% to 2.2%) decreased significantly during this time period (data not shown), which indicates increasing preterm and low birthweight twins survival. Consequently, these changes may have skewed cutoff values toward lower weights in the percentile charts. Unfortunately, we did not collect data with regard to environmental conditions of pregnant women including socioeconomic status, diet, or nutrition. Thus, we could not directly analyze the association between environmental changes and birthweight distributions. Secondly, like other population-based studies, our data is cross-sectional nature based on birth registration rather than longitudinal measurement of the same fetus over the course of gestation. Thus, preterm infants may be somewhat smaller that fetuses of the same gestational ages who remain in utero^36^. Thirdly, the relative small sample size at the lower limits of gestation age may decrease the validity of our results.

In summary, our study was the first to present percentile curves of birthweight by gestational age for Chinese twin neonates using a ten-year population-based dataset. Twin specific birthweight curves are important for obstetricians to predict the birth weights of twins. Pediatricians can use these curves to situate the birthweight of the individual twins by gender in relation to other twins. However, further research is required for the validation and implementation of twin birthweight curves into clinical practice. Only when this condition is satisfied, twins at risk of perinatal morbidity and mortality can be identified.

## Material and Methods

### Twin population

Wuhan is the capital and largest city of Hubei province in the center of mainland China (113°41′-115°05′, 29°58′-31°22′). The city has around 10.33 million permanent residents by the end of 2014 and covers 8494 km^2^. During 2006–2015, the population growth rate was 7.25‰, and birth rate in Wuhan was about 12.22‰.

Birth record data were obtained from the Wuhan Birth Registry Information System (WBRIS) affiliated to Bureau of Health and Family Planning Commission which was established in 2003. Since then, birth certificates of all live births have been directly reported to this database by obstetricians or well-trained midwives, as well as guardians. The WBRIS included all registered births from 95 hospitals within the Wuhan area, which covered almost 100% of all hospital-registered births. Considering the steadiness, we used data of new births from 2006 to 2015 in this study. During this period of time, records for 836,229 newborns, including 11,726 complete pairs of twins (23,452 individuals), were presented in WBRIS.

Each record in the database of WBRIS contained information on parent’s residential address, birth outcome (Live Birth, Still birth, Died within seven days after delivery, and presence of congenital anomalies), parity (Primary or Multiple), plurality (Singleton, Twin, Triplet, etc), modes of delivery (vaginal delivery or caesarean section), as well as infant’s sex, gestational age in complete weeks, birth weight, body length, chest circumference, and head circumference. Specifically, gestational age in complete weeks was determined primarily through last-menstrual-period (LMP) estimation, and estimates from the last ultrasound scan before delivery (usually at the second or third trimester). These two methods were cross-referenced when available. Stillbirths have been recorded in the WBRIS since 16 weeks of gestation. The birthweight of each neonate is measured twice to the nearest of 0.1 gram within 24-hour after the childbirth.

### Study Sample

**Figure 4:**
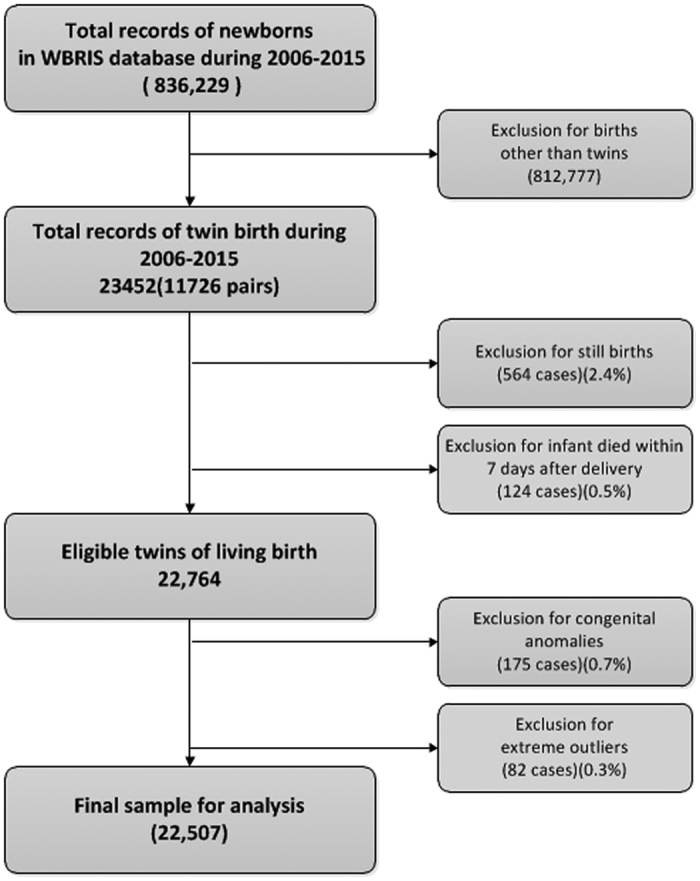
Flow of the participants. The total number of 111 twin births less than 26 weeks of gestational age were excluded, in that 96 stillbirths, 3 neonatal deaths and 12 alive outliers. WBRIS, Wuhan Birth Registry Information System.

The inclusion criteria for this study are twin births, a birthweight greater that 400 grams, and a gestational age at least 26 weeks. Only liveborn twin individuals were included in the preparation of birthweight percentiles. We excluded non-live twin births (stillbirth and those that died within seven days after delivery) (2.93%) and births with congenital anomalies (0.7%). Lastly, we excluded extreme birthweight outliers (0.3%) (defined as values three-time the standard deviation (SD) away from the mean of twin births born at the same gestational age in weeks). After all exclusion criteria, 22,507 births of twin individual remained in the data set, including 11,861 (52.7%) male and 10, 646 (47.3%) female twins. (See the Fig. 4 below).

### Ethical approval and informed consent

*The methods were carried out in “accordance” with the approved guidelines and regulation.* Ethical approval to conduct the research was obtained from the Research Ethics Committee of Wuhan Medical & Healthcare Center for Women and Children, Wuhan, China (NO. 2015-62). And informed consent was obtained from all subjects.

### Statistical analysis and creation of percentile curves

All data were analyzed by using SAS, version 9.2 (SAS Institute, Inc., Cary, NC, USA). The percentile values for birthweight were analyzed using gestational age by sex. Differences were considered to be significant at the 0.05.

The significance of differences in mean values of gestational age and birthweight was statistically analyzed using Students’ *t*-test for two groups and ANOVA for three groups or more. We estimated and presented in percentiles the distribution of birthweight for each 1-week increment of gestational age from 26 to 42 weeks (twin births born beyond 42 weeks of gestational age were combined into one group because of small sample size of each strata).

Furthermore, we plotted the curves along the 3^th^, 10^th^, 25^th^, 50^th^, 75^th^, 90^th^, 97^th^ percentiles by using the Cole’s Lambda Mu Sigma (LMS) method^37^. LMS approach initially estimates the three parameters of Box-Cox transformation of the distribution of the measurement. The L determines a nonlinear transformation of birthweight, such that its distribution approximates the normal distribution. The M stands for the mean of that normal distribution, and S for the coefficient of variation. The three parameters are constrained to change smoothly as the covariate changes. L, M, and S correspond to the following formulas: *Z* = [(X/M)^*L*^ − 1]/LS, where X indicates the measured value of birth weight; and the centile = M*(1 + L*S*Z_*α*_)^1/L^, where *Z*_*α*_is the *z*-score that corresponds to a given percentile (e.g. 3^rd^, 10^th^, 25^th^, 50^th^, 75^th^, 90^th^, and 97^th^). The *Z*-score is a measure of the distance in SDs of a sample from the mean. To create smoothed percentile charts for the 3^rd^, 10^th^, 25^th^, 50^th^, 75^th^, 90^th^, and 97^th^ percentiles, LMS Chart Maker light version 2.54 (Medical Research Council, London, UK) was used.

## Additional Information

**How to cite this article**: Zhang, B. *et al*. Birthweight percentiles for twin birth neonates by gestational age in China. *Sci. Rep.* **6**, 31290; doi: 10.1038/srep31290 (2016).

## Figures and Tables

**Table 1:** Birthweight and gestational age of twins according to variables.

Variables	No. of cases (%)	Mean of birthweight (g) (s.d.)	Gestational age (weeks) (s.d.)
Total of twin births	22507(100.0)	2384.8(489.1)	35.8(2.4)
Sex			
Male twin	11861(52.7)	2424.3(501.8)	35.8(2.4)
Female twin	10646(47.3)	2328.2(470.7)**	36.0(2.3)**
Like-sexed female	7178(31.9)	2334.2(459.6)	36.0(2.3)
Like-sexed male	8419(37.4)	2402.2(498.1)^a^,^b^	35.8(2.4)^a^
Unlike-sexed	6910(30.7)	2427.7(479.2)^a^,^b^	35.8(2.3)^a^
Method of delivery			
Caesarean section	20376(90.5)	2439.6(444.5)	36.1(2.02)
Virginal	2131(9.4)	1898.3(547.2)**	33.3(3.4)**
Birth order			
First	11329(50.3)	2423.5(480.7)	35.9(2.3)
Second	11178(49.7)	2353.2(480.5)**	35.9(2.3)
Parity			
Primipara	12891(57.3)	2385.2(476.6)	35.9(2.3)
Multipara	9616(43.7)	2393.2(488.8)	35.8(2.4)
Preterm birth (<37 weeks)			
Yes	12500(55.5)	2197.5(476.7)	34.3(2.0)
No	10007(44.5)	2627.3(368.5)**	37.7(1.0)**
Low birthweight (<2500g)			
Yes	11871(52.7)	2038.3(353.8)	34.9(2.5)
No	10636(47.3)	2780.8(245.1)**	37.0(1.4)**

^a^Means both unlike-sexed and like-sexed male compared to like-sexed female, *P* < 0.01.

^b^Means unlike-sexed compared to like-sexed male, *P* < 0.01; **P < 0.01.

**Table 2:** The birth weight curves L,M and S parameters and percentile birth weight by gestational age in female twin newborns in Wuhan from 2006 to 2015.

GA (wks)	No.	L	M	S	Percentiles						
					3^rd^	10^th^	25^th^	50^th^	75^th^	90^th^	97^th^
26	11	−0.29	980	15.45	741	809	885	980	1,089	1,201	1,327
27	14	−0.35	1040	15.15	793	862	941	1,040	1,153	1,271	1,404
28	64	−0.40	1130	15.20	862	937	1,023	1,130	1,254	1,384	1,531
29	70	−0.31	1260	16.85	931	1,023	1,128	1,260	1,413	1,575	1,759
30	142	−0.27	1400	17.35	1,024	1,128	1,249	1,400	1,576	1,760	1,970
31	217	−0.23	1560	17.67	1,133	1,251	1,388	1,560	1,759	1,968	2,204
32	326	0.15	1700	17.85	1,205	1,347	1,507	1,700	1,914	2,128	2,359
33	549	−0.15	1870	16.33	1,385	1,522	1,678	1,870	2,088	2,312	2,560
34	795	−0.07	2100	15.10	1,585	1,733	1,899	2,100	2,324	2,551	2,797
35	1,381	0.05	2260	13.97	1,735	1,888	2,058	2,260	2,481	2,700	2,934
36	2,212	0.11	2400	14.32	1,826	1,994	2,179	2,400	2,640	2,877	3,129
37	2,537	0.17	2550	13.24	1,977	2,147	2,332	2,550	2,785	3,014	3,254
38	1,410	0.17	2640	12.58	2,074	2,242	2,425	2,640	2,871	3,095	3,329
39	598	0.15	2685	12.41	2,117	2,286	2,469	2,685	2,916	3,142	3,377
40	236	0.14	2660	11.42	2,139	2,295	2,463	2,660	2,870	3,074	3,287
41	52	0.12	2630	11.11	2,129	2,279	2,441	2,630	2,832	3,028	3,233
42	32	0.13	2575	10.17	2,122	2,258	2,405	2,575	2,756	2,930	3,111

(n = 10,646). GA, gestational age (e.g. 36 weeks represent 36 weeks +0 day to +6 days).

LMS, Lambda Mu Sigma.

**Table 3:** The birth weight curves L,M and S parameters and percentile birth weight by gestational age in male twin newborns in Wuhan from 2006 to 2015.

GA (wks)	No.	L	M	S	Percentiles						
					3^rd^	10^th^	25^th^	50^th^	75^th^	90^th^	97^th^
26	17	−0.51	960	16.45	720	786	862	960	1,075	1,200	1,344
27	36	−0.35	1,100	15.15	838	912	996	1,100	1,220	1,345	1,485
28	92	−0.40	1,200	16.20	900	983	1,079	1,200	1,341	1,490	1,660
29	122	−0.31	1,360	16.85	1,005	1,104	1,217	1,360	1,526	1,700	1,898
30	186	−0.17	1,450	17.17	1,059	1,169	1,294	1,450	1,629	1,814	2,021
31	281	−0.23	1,610	17.30	1,177	1,297	1,436	1,610	1,811	2,021	2,258
32	415	−0.15	1,800	17.85	1,297	1,438	1,599	1,800	2,031	2,271	2,540
33	622	−0.15	2,000	16.33	1,482	1,628	1,794	2,000	2,233	2,473	2,738
34	856	−0.05	2,180	17.10	1,585	1,754	1,945	2,180	2,445	2,717	3,014
35	1,539	0.05	2,400	13.97	1,843	2,005	2,185	2,400	2,635	2,868	3,115
36	2,553	0.08	2,500	14.32	1,904	2,079	2,270	2,500	2,751	2,999	3,263
37	2,842	0.11	2,650	13.24	2,059	2,233	2,424	2,650	2,895	3,135	3,388
38	1,482	0.15	2,740	12.27	2,167	2,337	2,522	2,740	2,973	3,200	3,437
39	502	0.21	2,750	12.11	2,178	2,349	2,534	2,750	2,980	3,203	3,435
40	229	0.24	2,770	12.23	2,186	2,361	2,550	2,770	3,004	3,230	3,465
41	59	0.22	2,730	13.41	2,106	2,292	2,493	2,730	2,984	3,231	3,489
42	28	0.31	2,665	13.67	2,039	2,226	2,428	2,665	2,917	3,160	3,413

(n = 11,861). GA, gestational age (e.g. 36 weeks represent 36 weeks +0 day to +6 days).

LMS, Lambda Mu Sigma.
